# Kahweol activates the Nrf2/HO-1 pathway by decreasing Keap1 expression independently of p62 and autophagy pathways

**DOI:** 10.1371/journal.pone.0240478

**Published:** 2020-10-12

**Authors:** Hye-Young Seo, So-Hee Lee, Ji-Ha Lee, Jae Seok Hwang, Mi Kyung Kim, Byoung Kuk Jang

**Affiliations:** Department of Internal Medicine, School of Medicine, Institute for Medical Science, Keimyung University, Daegu, South Korea; Augusta University, UNITED STATES

## Abstract

Kahweol is a diterpene found in coffee beans and unfiltered coffee drinks. Several studies have demonstrated that kahweol induces the nuclear factor erythroid-2 related factor 2/ hemeoxygenase-1 (Nrf2/HO-1) pathway; however, the mechanisms involved are currently unknown. Kelch-like ECH-associated protein 1 (Keap1) is a major regulator of Nrf2 expression and is degraded mostly by autophagy. The p62 protein enhances binding to Keap1 and contributes to the activation of Nrf2. Here, we examined the role of Keap1 regulation in the effect of kahweol on the Nrf2/HO-1 pathway in hepatocytes. In AML12 cells and primary mouse hepatocytes, kahweol increased the levels of Nrf2 and HO-1 protein without increasing expression of the Nrf2 mRNA. In addition, kahweol reduced Keap1 protein levels significantly without decreasing Keap1 mRNA levels. Although regulation of the Keap1-Nrf2-pathway by p62-dependent autophagy is well known, we confirmed here that the reduction of Keap1 protein levels by kahweol does not involve p62-dependent autophagy degradation or ubiquitination. In conclusion, kahweol increases the expression of Nrf2 in hepatocytes by inhibiting translation of the Keap1 mRNA.

## Introduction

As the main detoxification organ, the liver maintains metabolic homeostasis under normal conditions. However, certain drugs, infections, external exposures, and tissue damage can increase the amount of reactive oxygen species (ROS) in the liver [1–2]. Excessive ROS can interfere with liver homeostasis and cause oxidant stress, which plays an important role in the development of diseases such as viral hepatitis, nonalcoholic fatty liver disease, alcoholic liver disease, and drug-induced liver injury [3–6]. Oxidative stress activates the hepatic antioxidant defense system. The nuclear factor erythroid-2 related factor 2 (Nrf2)/Kelch-like ECH-associated protein 1 (Keap1) pathway plays a central role in protecting cells against oxidative stress. Under stress conditions, Nrf2 dissociates from Keap1 and enters the nucleus, where it regulates the expression of antioxidant genes such as hemeoxygenase-1 (HO-1) [7, 8]. SQSTM1 (also known as p62) also binds to Keap1 and contributes to the activation of Nrf2 [9]. Therefore, the p62-Keap1-Nrf2 signaling pathway plays an important role in the cellular response to oxidative stress. Previous studies have shown that Nrf2 prevents oxidative stress and injury in the liver by activating cytoprotective genes [10, 11]. Thus, Nrf2 activators have potential role as therapeutic agents against oxidative stress during liver injury [12].

Kahweol, a diterpene present in coffee, has a variety of pharmacological actions, including anti-inflammatory, liver protection, anti-cancer, anti-diabetes, and anti-osteocyte formation effects [13–16]. Recently, our study reported the protective effects of kahweol on liver fibrosis and liver inflammation [17, 18]. In addition, kahweol has antioxidant effects in the liver [19, 20], and this effect may be mediated at least in part by Nrf2 [21]. Therefore, in this study, we examined the mechanism of Nrf2 activation by kahweol and its antioxidant effect in the liver.

## Materials and methods

### Chemicals

Kahweol was purchased from LKT Laboratories Inc. (St. Paul, MN, USA) and hydrogen peroxide solution (H_2_O_2_) was purchased from Sigma-Aldrich (St. Louis, MO, USA). The anti-Keap1 antibody was purchased form Proteintech (Rosemont, IL, USA), the anti-Nrf2 (1:2000) antibody was purchased from Thermo Scientific (Waltham, MA, USA), the anti-p62 (1:10000) and Anti-ATG5 (1:2000) antibody antibody was purchased from Abcam (Cambridge, UK), and the anti-HO-1 (1:5000) antibody was purchased from Santa Cruz Biotechnology (Dallas, TX, USA). The anti-ATG7 (1:2000), anti-β-tubulin (1:10000), anti-cleaved caspase 3 (1:2000), anti-GAPDH, Anti-ubiquitin (1:2000) and anti-Beclin1 (1:2000) antibodies were purchased from Cell Signaling Technology (Beverly, MA, USA).

### Cell culture

The AML12 mouse hepatocyte cell line was purchased from the American Type Culture Collection (Manassas, VA, USA). The cells were maintained in 5% CO2 and 95% air at 37°C and were cultured in DMEM/F12 (GIBCO-BRL, Grand Island, NY, USA) supplemented with insulin-transferrin-selenium (GIBCO-BRL), dexamethasone (40 ng/mL; Sigma-Aldrich), antibiotics (GIBCO-BRL), and 10% fetal bovine serum (FBS) (Hyclone, Logan, UT, USA). The cells were serum-starved in medium containing 0.5% FBS and then treated with or without kahweol.

### Measurement of ROS production

ROS production in AML12 cells was measured using the fluorescent dye H_2_DCFDA (Thermo Scientific). After treatment with 1 mM H_2_O_2_ for 5 h, the cells were incubated with 5 μM H_2_DCFDA for 20 min and observed using a fluorescent microscope (Carl Zeiss, Thornwood, NY, USA).

### Cell viability assay

Cell viability was detected by Cell Counting Kit-8 (CCK-8, Dojindo, Kumamoto, Japan) assay. AML12 cells seeded on a 96-well plate (1 x 10^4^ cells/100 μL/well) for 24 h, and then untreated (control) or treated with H_2_O_2_ or H_2_O_2_ plus kahweol. Subsequently, 10 μL of CCK-8 solution was added to each well and the cells were incubated for an additional 2 h. Finally, the absorbance was measured at 450 nm on a spectrophotometer.

### Flow cytometry analysis

AML12 cells were suspended in 100 μL of PBS, and then ethanol (200 μL) was added while vortexing. Subsequently, the cells were incubated at 4°C for 1 h, washed with PBS, resuspended in 250 μL of 1.12% sodium citrate buffer containing 12.5 μg of RNase, and then incubated at 37°C for a further 30 min. The cellular DNA was then stained by applying 250 μL of propidium iodide (50 μL/mL) for 30 min. The stained cells were analyzed by fluorescence-activated cell sorting on a FACScan flow cytometer and the relative DNA content was determined based on the red fluorescence intensity.

### Isolation of primary hepatocytes

Male 6-8-week-old C57BL/6 mice were purchased from Central Lab Animal (Seoul, Korea) and housed in a facility under a 12 h light/dark cycle. All experiments were approved by the Institutional Animal Care and Use Committee of Keimyung University (KM-2017-25R1). All animal procedures were performed following the institutional guidelines for animal research. Hepatocytes were isolated from C57BL/6 mice by perfusing the liver through the portal vein. The liver was perfused with resuspension buffer (5.4 mM KCl, 0.44 mM KH2PO4, 140 mM NaCl, 0.34 mM Na2HPO4, 0.5 mM EGTA, and 25 mM Tricine, pH 7.2) at a rate of 5 mL/min for 10 min, and then with collagenase solution [Ca2+- and Mg2+-free Hanks Balanced Salt Solution, pH 7.2, containing 0.75 mg/mL collagenase type I (Worthington Biochemical Corp., Freehold, NJ, USA)] at a rate of 5 mL/min for 10 min. After perfusion, the liver was shaken for 20 min at 37°C, filtered through a 70 μm nylon mesh, and then centrifuged at 42 × g for 5 min at 4°C. The pelleted hepatocytes were re-suspended in William’s Medium E (GIBCO-BRL) and seeded onto type I collagen-coated 60 mm dishes (IWAKI Scitech Kiv, Tokyo, Japan). The viability of the hepatocytes was always higher than 85%. After incubation for 2–3 h, the medium was replaced with Medium 199 (Sigma-Aldrich). Hepatocytes were treated with or without kahweol in 0.5% FBS, and then processed for isolation of proteins and RNA as described below.

### Small interfering RNA (siRNA)-mediated depletion of p62

A pre-designed siRNA targeting p62 (siRNA-p62) and a scrambled control siRNA (siCon) were purchased form Snata Cruz Biotechnology. Cells were transfected with 100 nM siRNA using Lipofectamine RNAiMAX (Invitrogen, Carlsbad, CA, USA) for 5 h, cultured in medium containing 0.5% FBS, and harvested ~48 h after transfection.

### Western blot analysis

The cells were harvested in RIPA buffer (Thermo Scientific) containing protease/phosphatase inhibitors (Inhibitor Cocktail solution; genDEPOT, Katy, TX, USA) for 30 min at 4°C. Mouse liver samples were homogenized and lysed in RIPA buffer. Protein concentrations were determined using the BCA assay (Thermo Scientific). Equal amounts of solubilized proteins were separated by SDS-PAGE and transferred to PVDF membranes (Millipore, Billerica, MA, USA). The membranes were sequentially incubated in blocking buffer (5% skimmed milk prepared in Tris-buffered saline containing 0.1% Tween 20), primary antibodies, and then appropriate horseradish peroxidase-conjugated secondary antibodies. Signals were visualized using the Clarity™ Western ECL substrate kit (Bio-Rad, Richmond, CA, USA). The membranes were re-probed with an anti-GAPDH or anti-tubulin antibody to verify that an equal amount of protein had been loaded in each lane. Signal intensities were quantitated by densitometry using ImageJ software (version 1.52a) (NIH, Bethesda, MD, USA).

### Immunoprecipitation

Total protein (500 μg) immunoprecipitated by shaking with the anti-Keap1 primary antibody overnight, followed by the addition of a protein G-agarose bead suspension (30 μl) with additional shaking for 3 h. After centrifugation at 3,000 rpm for 1 min, the supernatant was discarded and the immunoprecipitated beads were collected and added with cell lysis buffer. Following a final wash step, the immunoprecipitate was resuspended in 30 μl of sample buffer and boiled for 5 min. Subsequently, a 20 μl aliquot of supernatant from each sample was loaded onto an SDS-PAGE gel.

### Quantitative real-time RT-PCR

Total RNA was isolated from cells and tissue extracts using TRIzol reagent (Invitrogen). Reverse transcription was performed using the Maxima First Strand cDNA Synthesis Kit (Thermo Scientific). Quantitative real-time RT-PCR was performed using a SYBR Green PCR Master Mix Kit (Roche Diagnostics, Indianapolis, IN, USA) and a CFX Connect Real-Time PCR system (Bio-Rad). The PCR conditions were as follows: 45 cycles of 95°C for 30 s, 60°C for 10 s, and 72°C for 15 s. GAPDH was used as an internal standard. The primer sequences were as follows: mouse Nrf2 forward, 5’-AAAGCACAGCCAGCACATTC-3’, and reverse, 5’-AATGGGGCTTTTTGATGACC-3’; mouse HO-1 forward, 5’-TACCTTCCCGAACATCGACA-3’, and reverse, 5’-TCTGCAGGGGCAGTATCTTG-3’; mouse p62 forward, 5’-AAGTTCCAGCACAGGCACAG-3’, and reverse, 5’-CTCCTCCTGAGCAGTTTCCC-3’; and mouse GAPDH forward, 5’- ACGACCCCTTCATTGACCTC-3’, and reverse, 5’-ATGATGACCCTTTTGGCTCC-3’.

### Atg7^flox/flox^-albumin Cre mice (Atg7^f/f^ Alb-Cre)

Atg7 floxed mice (Atg7^f/f^) were bred with albumin-Cre mice to generate hepatocyte-specific Atg7 knockout mice (Atg7^f/f^ Alb-Cre^+^). The mice were a kind gift from Dr. Myung-Shik Lee (Yonsei University), with the permission of Dr. Masaaki Komatsu (Tokyo Metropolitan Institute of Medical Science). All experiments were approved by the Institutional Animal Care and Use Committee of Keimyung University (KM-2016-28). All animal procedures were performed in accordance with the institutional guidelines for animal research.

### Statistical analysis

Data are expressed as the mean ± SEM. Statistical analyses were performed with one-way ANOVAs with Bonferonni correction and p < 0.05 was considered statistically significant. All experiments were performed at least three times.

## Results

### Kahweol suppress H_2_O_2_-induced ROS generation and apoptosis in hepatocytes

**Fig 1:**
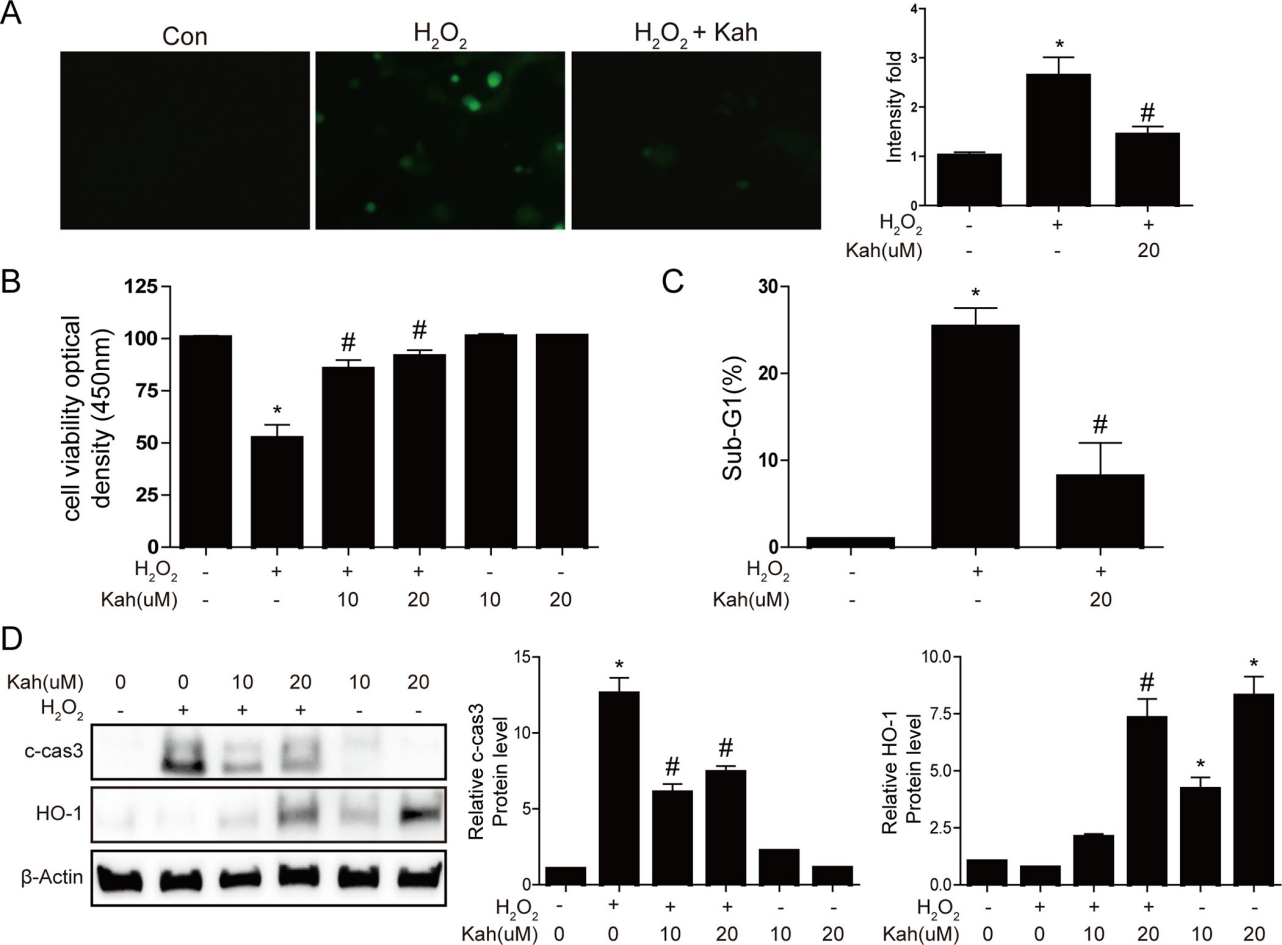
The effects of kahweol on H_2_O_2_-induced oxidant stress in hepatocytes. AML12 cells were treated with kahweol (10 or 20 μM) for 19 h and then incubated with H_2_O_2_ (1mM) for a further 5 h. (**A**) DCF-DA staining of cells treated with 1 mM H_2_O_2_ and with or without 10 μM kahweol. Data in the bar graph are means ± SEM of three independent measurements. * *p* < 0.05 compared with the control, ^#^ *p* < 0.05 compared with the H_2_O_2_ treatment. (B) The viability of AML12 cells treated with or without H_2_O_2_ and kahweol, as determined by a CCK-8 assay. (C) FACScan flow cytometry analyses of AML12 cells treated with or without H_2_O_2_ and kahweol. Apoptosis was determined based on the sub-G1 population. (**D**) Western blot analyses of cleaved caspase 3 and HO-1 levels in AML12 cells treated with or without kahweol (10 or 20 μM) and 1 mM H_2_O_2_. * *p* < 0.05 compared with the control, ^#^ *p* < 0.05 compared with the H_2_O_2_ treatment. Con, control; Kah, kahweol.

First, we investigated whether kahweol protects AML12 cells against H_2_O_2_-induced ROS generation and apoptosis. Incubation of the cells with kahweol (10 or 20 μM) reduced H_2_O_2_-induced ROS production significantly (*p <* 0.05, Fig 1A). Furthermore, treatment with H_2_O_2_ reduced the viability of AML12 cells significantly, but treatment with kahweol prevented this decrease (p < 0.05, Fig 1B). A flow cytometry analysis revealed that H_2_O_2_ induced an accumulation of the sub-G1 population, an effect that was prevented by treatment with kahweol (*p <* 0.05, Fig 1C). In addition, western blotting revealed that kahweol increased the expression of HO-1 and attenuated the H_2_O_2_-induced expression of cleaved caspase 3 significantly in AML12 cells (*p <* 0.05, Fig 1D). Overall, these results suggest that kahweol has antioxidant and anti-apoptotic effects in hepatocytes.

### Kahweol increases the levels of Nrf2 and HO-1 in hepatocytes

**Fig 2:**
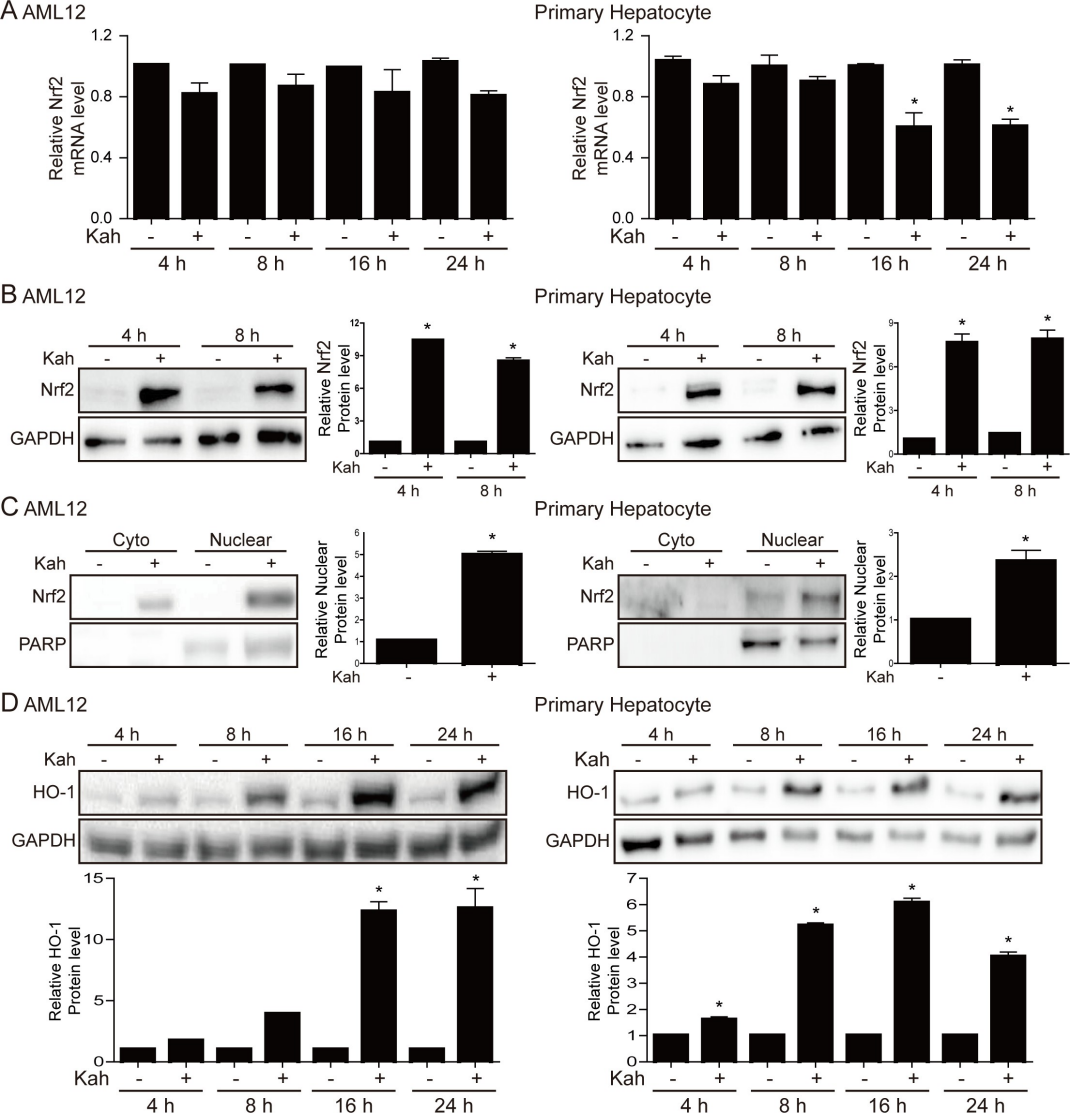
The effects of kahweol (Kah) on Nrf2 and HO-1 expression. AML12 cells were treated with 20 μM kahweol and primary hepatocytes were treated with 40 μM kahweol. (**A**) Representative real-time RT-PCR analyses of Nrf2 mRNA expression in AML12 cells (left) and primary hepatocytes (right). * *p* < 0.05, * *p* < 0.05 compared with the control for each time. (**B**) Western blot analyses showing the effect of kahweol on Nrf2 expression in whole cell extracts of AML12 cells (left) and primary hepatocytes (right). Data in the bar graph are means ± SEM of three independent measurements. * *p* < 0.05 compared with the control for each time. (**C**) Western blot analysis of cytoplasmic and nuclear extracts of AML12 cells (left) and primary hepatocytes (right), showing the effects of kahweol on Nrf2 expression. Kahweol was treated for 24 hours. Data in the bar graph are means ± SEM of three independent measurements. * *p* < 0.05 compared with the control. (**D**) Western blot analyses showing the effect of kahweol on HO-1 expression in AML12 cells (left) and primary hepatocytes (right). Data in the bar graph are means ± SEM of three independent measurements. * *p* < 0.05 compared with the control for each time.

Next, we examined changes in the expression level of Nrf2, which plays an important role in oxidative stress in kahweol-treated hepatocytes. Although kahweol did not affect the expression level of the Nrf2 mRNA in AML12 cells, it increased Nrf2 protein expression in both AML12 cells and primary hepatocytes (*p <* 0.05, Fig 2A and 2B). In addition, kahweol treatment increased Nrf2 nuclear translocation (*p <* 0.05, Fig 2C) and HO-1 protein expression (*p <* 0.05, Fig 2D) in both cell types.

### Kahweol decreases Keap1 protein expression in hepatocytes

**Fig 3:**
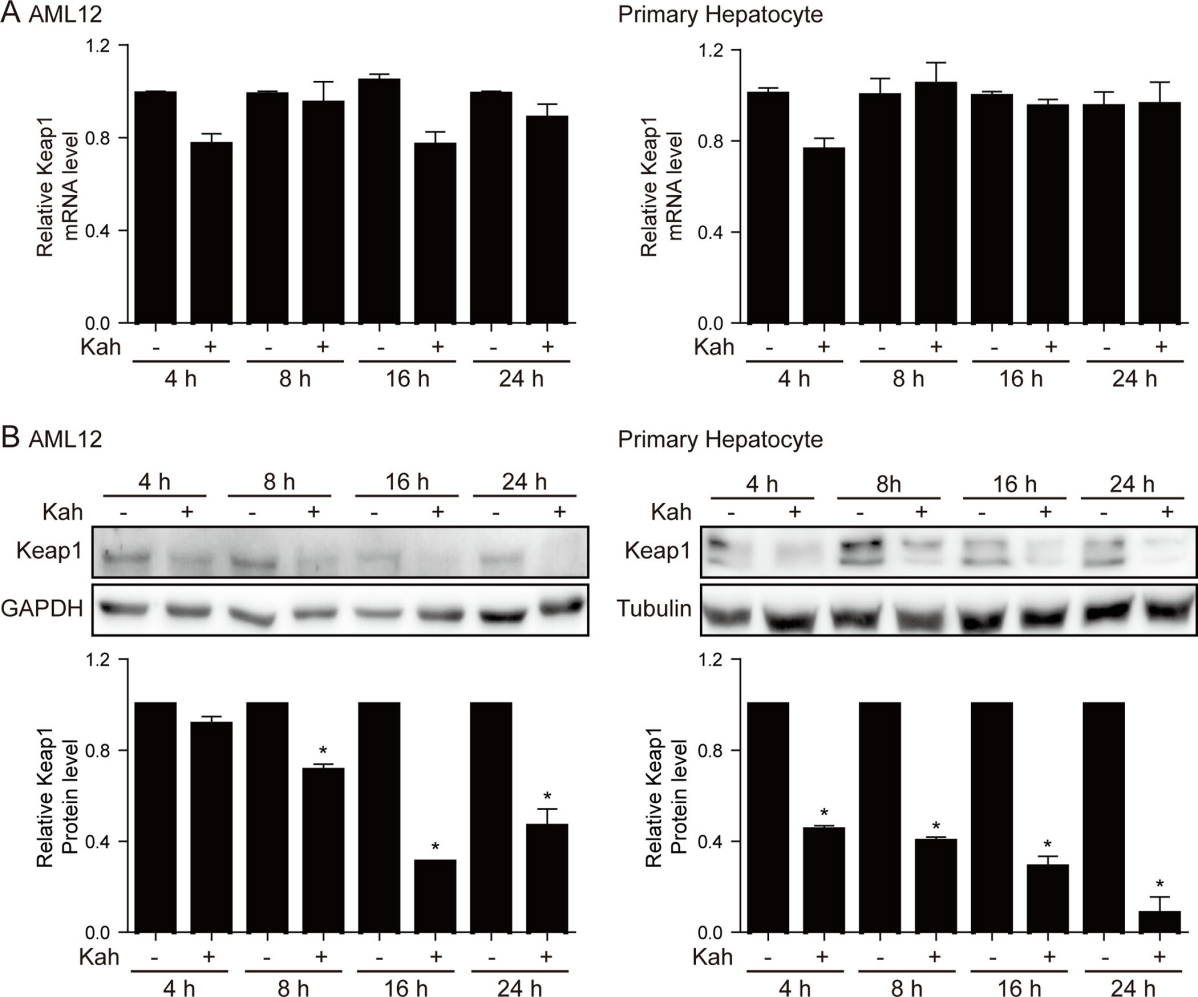
The effect of kahweol (Kah) on Keap1 expression. AML12 cells were treated with 20 μM kahweol and primary hepatocytes were treated with 40 μM kahweol. (**A, B**) Real-time RT-PCR (**A**) and western blot (**B**) analyses of Keap1 mRNA and protein levels, respectively, in AML12 cells (left) and primary hepatocytes (right). Data in the bar graphs are means ± SEM of three independent measurements. * p < 0.05 compared with the control for each time.

**Fig 4:**
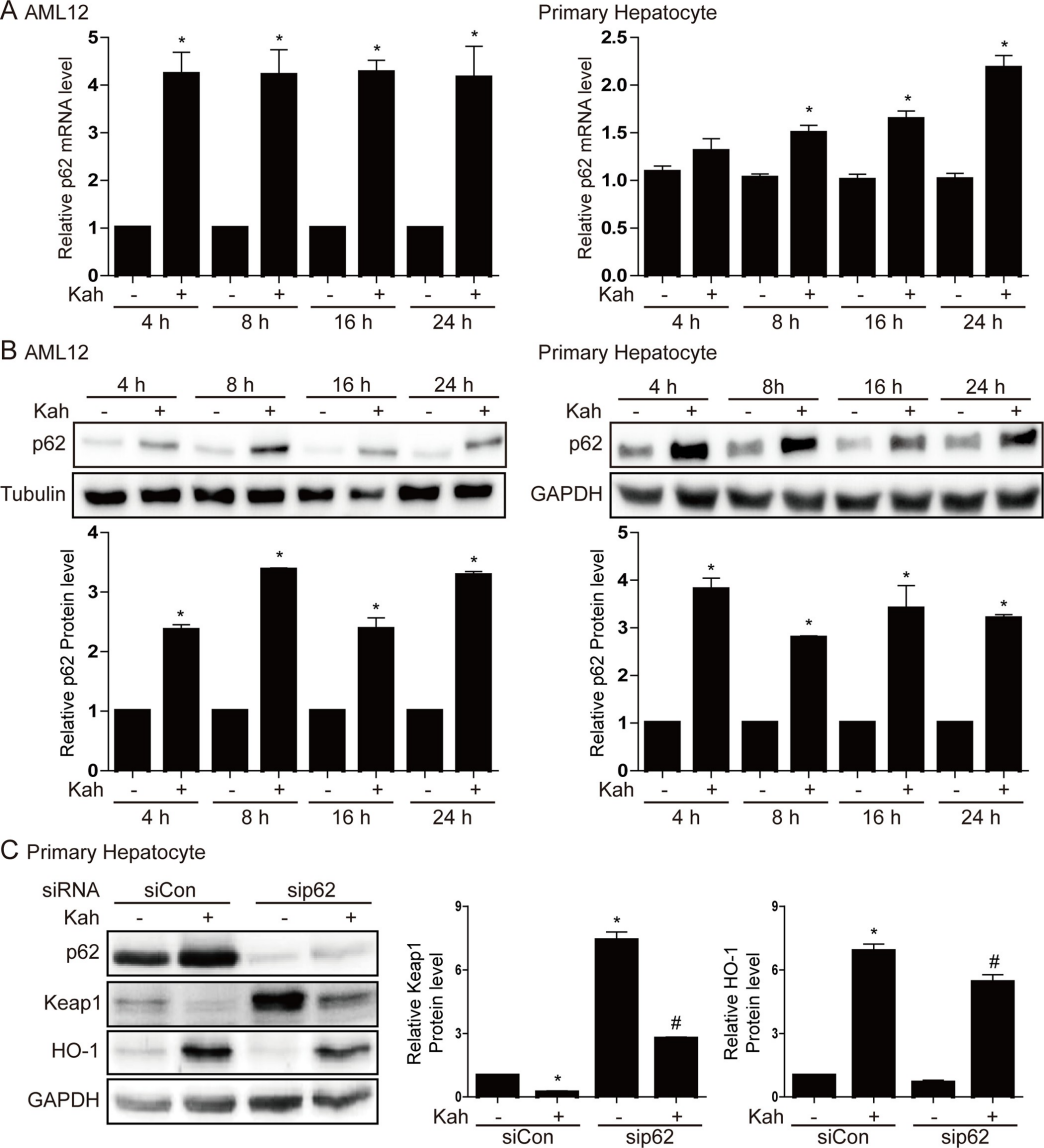
The effect of kahweol (Kah) on p62 expression. AML12 cells were treated with 20 μM kahweol and primary hepatocytes were treated with 40 μM kahweol. (**A,B**) Real-time RT-PCR (**A**) and western blot (**B**) analyses of p62 mRNA and protein levels, respectively, in AML12 cells (left) and primary hepatocytes (right). Data in the bar graph are means ± SEM of three independent measurements; * p < 0.05 compared with the control for each time. (**C**) Primary hepatocytes were transfected with a p62-specific siRNA (siRNA-p62; 100 nM) or a control siRNA (siCon; 100 nM), treated with 40 μM kahweol for 24 h, and then analyzed by western blotting. Data in the bar graph are means ± SEM of three independent measurements. * p < 0.05 compared with the control siRNA. ^#^ p < 0.05 compared with the siRNA-p62.

The Nrf2/HO-1 pathway is regulated by Keap1 and p62 [9, 22]; therefore, we investigated whether kahweol affects the expression of these two proteins in AML12 cells and primary mouse hepatocytes. In both cell types, kahweol reduced the Keap1 protein level significantly without affecting that of the mRNA (*p <* 0.05, Fig 3A and 3B). In addition, kahweol increased the levels of the p62 mRNA and protein significantly (*p <* 0.05, Fig 4A and 4B). When p62 binds to the Keap1, Nrf2 dissociates from the Keap1-Nrf2 complex and is thereby activated. However, despite siRNA-mediated suppression of p62 expression, kahweol was still able to increase HO-1 protein expression and decrease that of Keap1 (*p <* 0.05, Fig 4C). This finding suggests that increased levels of p62 do not play a role in kahweol-mediated increases in Nrf2/HO-1 expression and that kahweol activates Nrf2 by reducing Keap1 protein expression.

### The effect of kahweol on the degradation of Keap1

**Fig 5:**
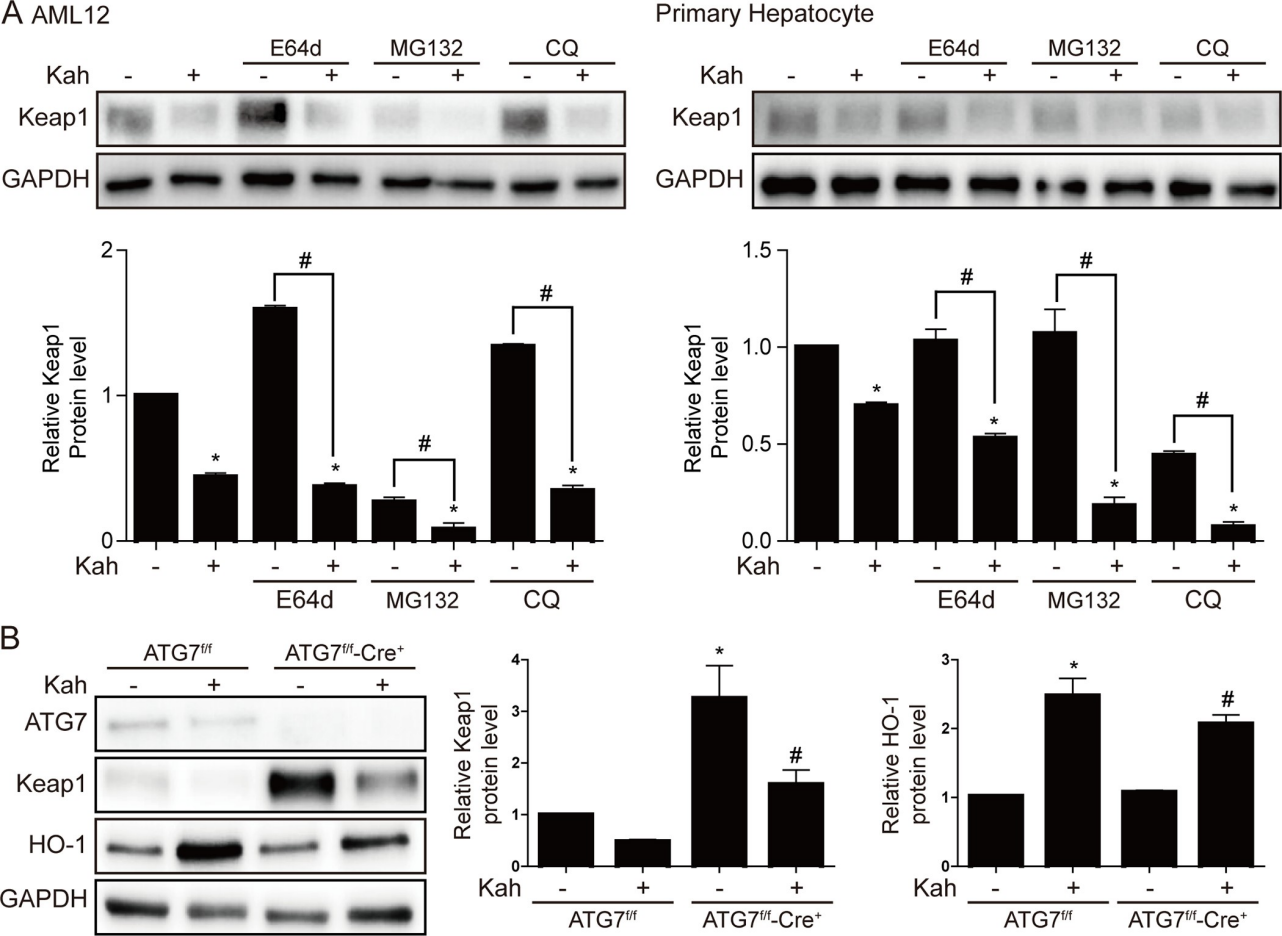
The effect of kahweol (Kah) on Keap1 protein degradation. (**A**) AML12 cells (left) were treated with 20 μM kahweol and primary hepatocytes (right) were treated with 40 μM kahweol in the absence or presence of E64d (10 μM), MG132 (0.5 μM), or chloroquine (CQ; 10 μM). Data in the bar graph are means ± SEM of three independent measurements. * p < 0.05 compared with the control. ^#^ p < 0.05. (**B**) Western blot analyses of Keap1 and HO-1 protein levels, in primary hepatocytes from ATG7^f/f^ Alb-Cre^+^ (autophagy-deficient) mice. Data in the bar graph are means ± SEM of three independent measurements. * p < 0.05 compared with the ATG7^f/f^, ^#^ p < 0.05 compared with the ATG7^f/f^-Cre^+^.

Recent studies show that Keap1 expression is reduced by autophagy or proteasome degradation [23, 24]. However, in our current study, kahweol had no significant effect on the expression levels of ubiquitin or autophagy-related markers (S1 Fig). In addition, even after the treatment of AML12 cells and primary mouse hepatocytes with a proteasome inhibitor (MG132) or autophagy inhibitors (E64d and chloroquine), kahweol was still able to reduce Keap1 protein expression in both cell types (*p <* 0.05, Fig 5A). To further investigate whether the kahweol-mediated reduction in Keap1 protein levels is dependent on autophagy, hepatocyte-specific ATG7-knockout mice were used. Kahweol still reduced Keap1 protein expression and increased HO-1 protein expression in primary hepatocytes from these autophagy-deficient mice (*p <* 0.05, Fig 5B), indicating that kahweol decreases Keap1 expression independently of the pathway that degrades the protein.

## Discussion

Here, we found that kahweol increased the levels of Nrf2 and HO-1 in liver cells, indicating an antioxidant effect. In addition, kahweol increased the level of the Nrf2 protein by decreasing Keap1 protein expression. Our results demonstrate that kahweol reduces Keap1 protein expression independently of p62-dependent autophagy degradation or ubiquitination; therefore, kahweol is thought to regulate translation of the Keap1 mRNA.

Disruption of the oxidation/antioxidation balance in the liver leads to an increase in the ROS level and consequently oxidative stress [25], a pathological mechanism that contributes to the progression of liver damage [1]. As a central regulator of antioxidant stress defenses, Nrf2 can activate antioxidant genes and protect liver cells against oxidative stress [8, 26]. Dysregulation or deficiency of Nrf2 in the liver is associated with the development of liver diseases, including hepatitis, fibrosis and, hepatocarcinogenesis [27–30]. Therefore, several studies have examined the abilities of antioxidants targeting Nrf2 to improve various liver diseases [31, 32].

Kahweol is a natural diterpene extracted from coffee beans that has antioxidant effects in the liver [16]. Specifically, kahweol regulates intracellular levels of ROS by increasing Nrf2 expression [21, 33]. Hwang et al. demonstrated that kahweol induces HO-1 through PI3K and p38/Nrf2 signaling in neuronal cells [21], and Fürstenau et al. showed that kahweol has a mitochondrial protective effect in neuronal cells by activating Nrf2/HO-1 [33]. However, the mechanisms involved in the antioxidant effects of kahweol in the liver are unknown. Here, kahweol increased Nrf2 protein expression in hepatocytes without altering the Nrf2 mRNA level, suggesting that the effect may have been caused by stabilization of the Nrf2 protein.

The p62 protein is a positive regulator of the Nrf2 pathway, and several studies have shown that increased p62 levels stabilize Nrf2 due to competition between p62 and Nrf2 for binding to Keap1 [9, 34, 35]. It has also been reported that p62-dependent autophagic degradation of Keap1 induces Nrf2 activation [36, 37]; however, in our current study, the induction of p62 by kahweol did not underlie the effects of kahweol on the Nrf2/HO-1 pathway. Indeed, when p62 was suppressed by siRNA-p62, kahweol was still able to reduce Keap1 protein expression and increase HO-1 protein expression in hepatocytes.

Downregulation of Keap1 protein expression is mediated by proteasome or autophagy degradation [23, 24]. Notably, Keap1 accumulates in the Atg7-deficient mouse liver, highlighting its degradation through autophagy [24, 38]. However, in this study, kahweol treatment of AML12 cells and primary mouse hepatocytes did not affect the expression levels of ubiquitin or autophagy markers, and kahweol reduced Keap1 protein levels in the presence of a proteasome or autophagy inhibitor. Therefore, the observed kahweol-mediated reduction in Keap1 levels was not due to protein`s degradation, suggesting that kahweol inhibits translation of the Keap1 mRNA.

In conclusion, our results demonstrate that kahweol increases Nrf2 expression by reducing Keap1 protein expression in a manner that is independent of p62-dependent autophagy degradation, resulting in antioxidant and anti-apoptotic effects in the liver. We suggest that kahweol inhibits translation of the Keap1 mRNA, although further studies are needed to confirm this hypothesis.

## Supporting information

S1 Fig The effects of kahweol (Kah) on ubiquitin and autophagy expression. AML12 cells were treated with 20 μM kahweol and primary hepatocytes were treated with 40 μM kahweol. (A) Western blot analyses showing the effect of kahweol on ubiquitin expression in AML12 cells (left) and primary hepatocytes (right). (B) After treatment of AML12 cells with kahweol for 24 h, proteins (500 μg per sample) were immunoprecipitated (IP) with an anti-Keap1 antibody and visualized by western blotting (WB) with anti-Keap1 and anti-ubiquitin antibodies. (C) Western blot analyses showing the effect of kahweol on LC3, ATG5, ATG7, and Beclin1 expression in AML12 cells (left) and primary hepatocytes (right).
(TIF)

S1 Raw images (PDF)
