# Supplementary figures and images for: Kahweol activates the Nrf2/HO-1 pathway by decreasing Keap1 expression independently of p62 and autophagy pathways
Source: PLoS One. 2020 Oct 12;15(10):e0240478. doi: 10.1371/journal.pone.0240478 (PMC7549774; doi:10.1371/journal.pone.0240478)

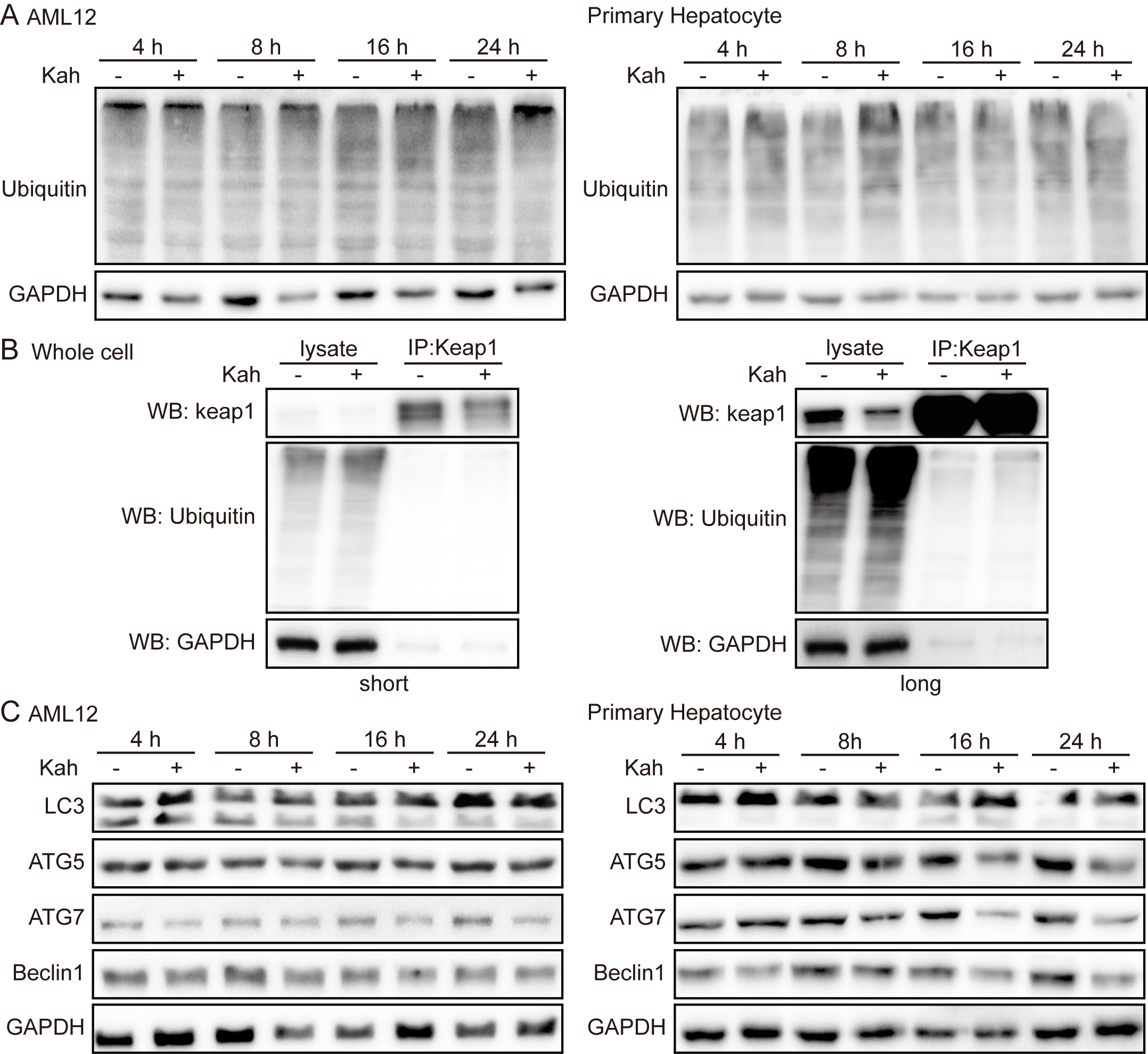

Supplement: S1 Fig — AML12 cells were treated with 20 μM kahweol and primary hepatocytes were treated with 40 μM kahweol. (A) Western blot analyses showing the effect of kahweol on ubiquitin expression in AML12 cells (left) and primary hepatocytes (right). (B) After treatment of AML12 cells with kahweol for 24 h, proteins (500 μg per sample) were immunoprecipitated (IP) with an anti-Keap1 antibody and visualized by western blotting (WB) with anti-Keap1 and anti-ubiquitin antibodies. (C) Western blot analyses showing the effect of kahweol on LC3, ATG5, ATG7, and Beclin1 expression in AML12 cells (left) and primary hepatocytes (right). (TIF) [file pone.0240478.s001.tif]
