# Supplementary material for: Kahweol activates the Nrf2/HO-1 pathway by decreasing Keap1 expression independently of p62 and autophagy pathways
Source: PLoS One. 2020 Oct 12;15(10):e0240478. doi: 10.1371/journal.pone.0240478 (PMC7549774; doi:10.1371/journal.pone.0240478)
Supplement: S1 Raw images — (PDF) [file pone.0240478.s002.pdf]

Figure 1D

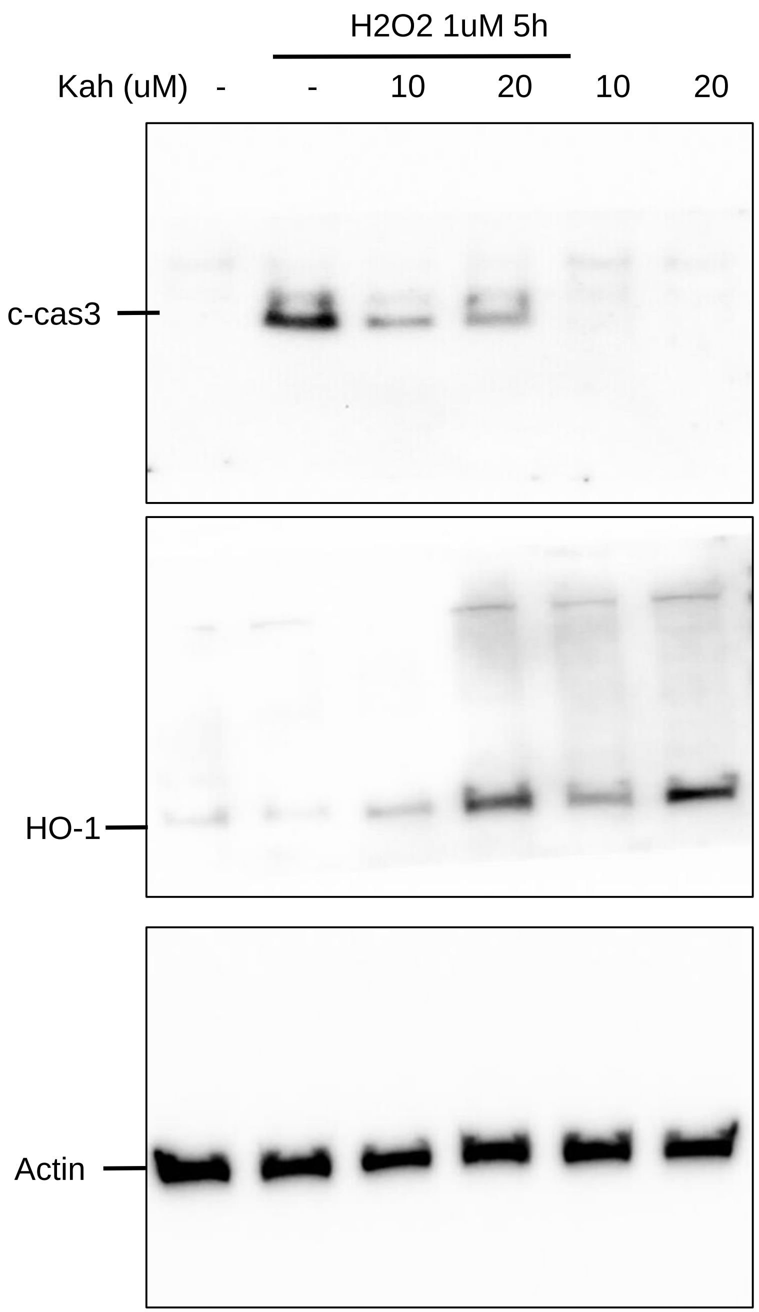

Figure 2B

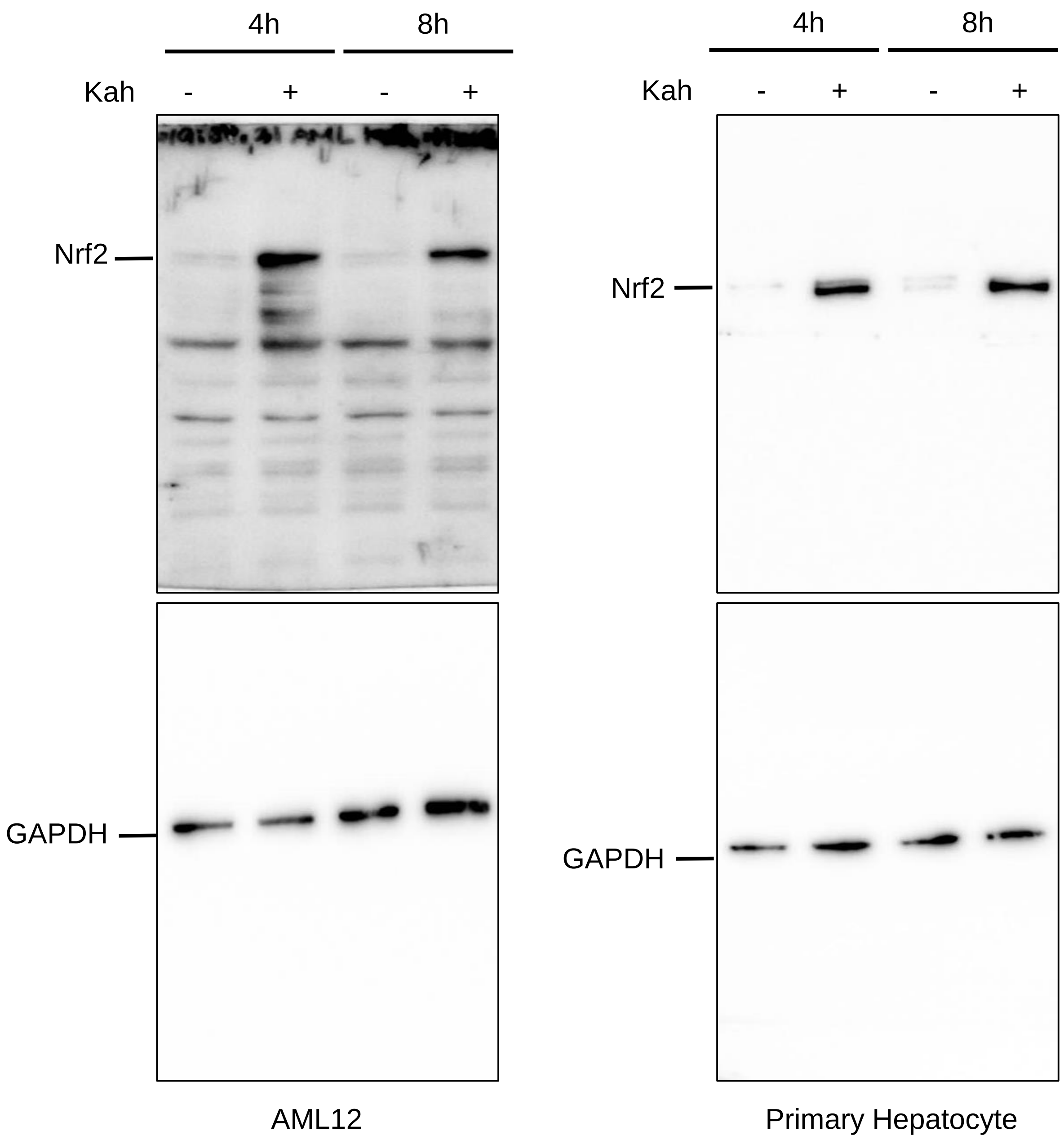

Figure 2C

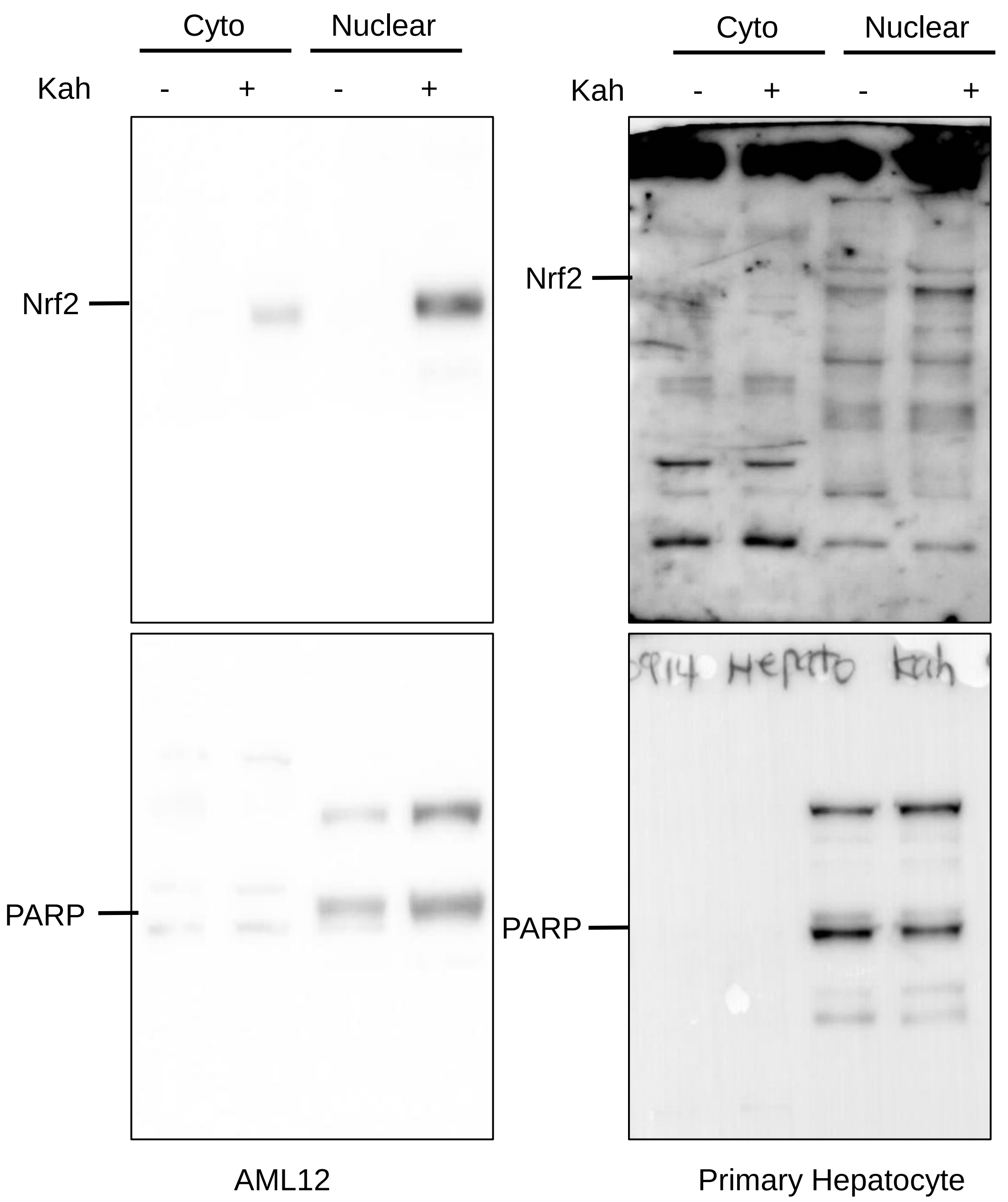

Figure 2D

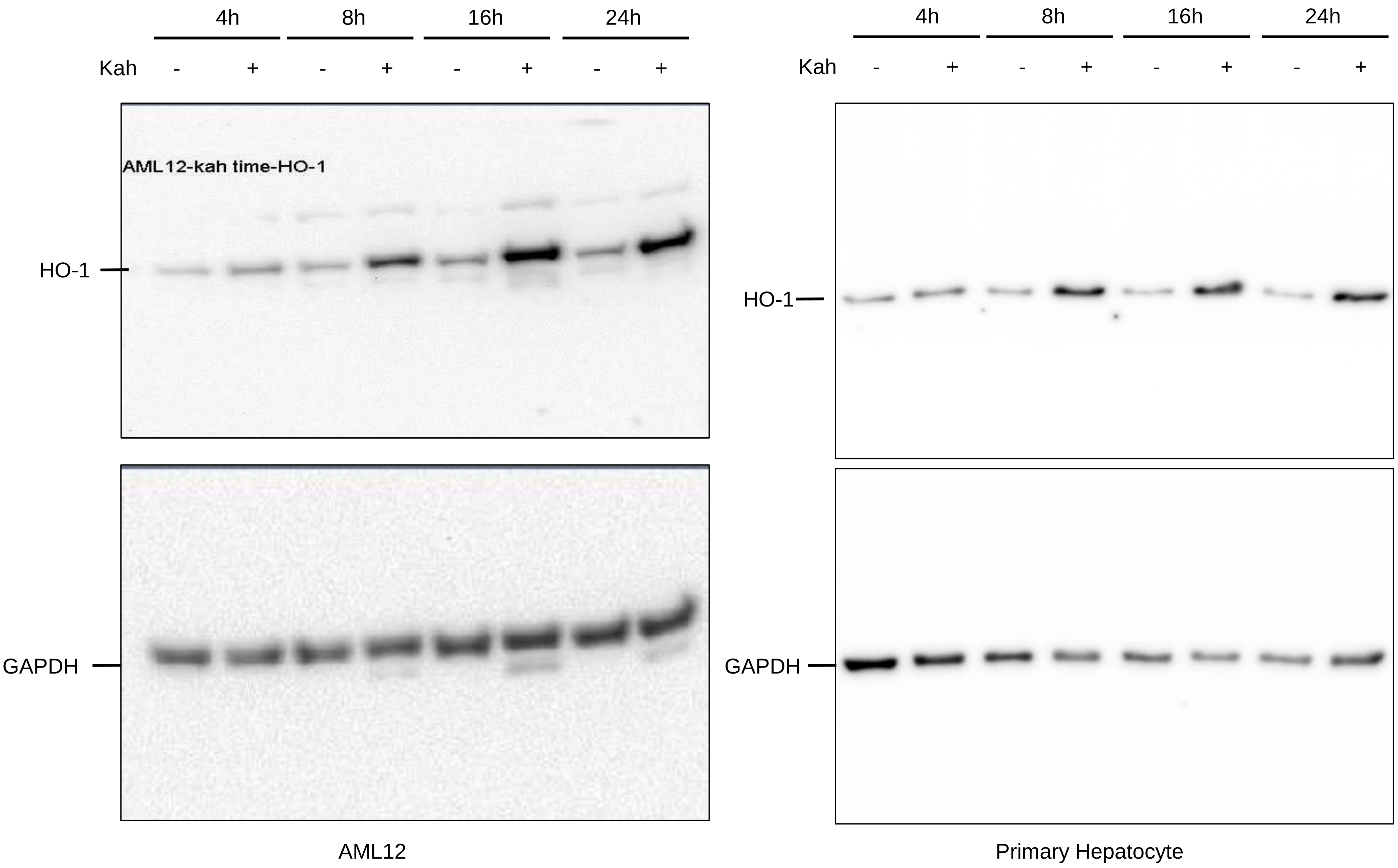
4h
8h
16h
24h
Kah - + - + - + - +
AML12-kah time-HO-1
HO-1
GAPDH
AML12
4h
8h
16h
24h
Kah - + - + - + - +
HO-1
GAPDH
Primary Hepatocyte

Figure 3B

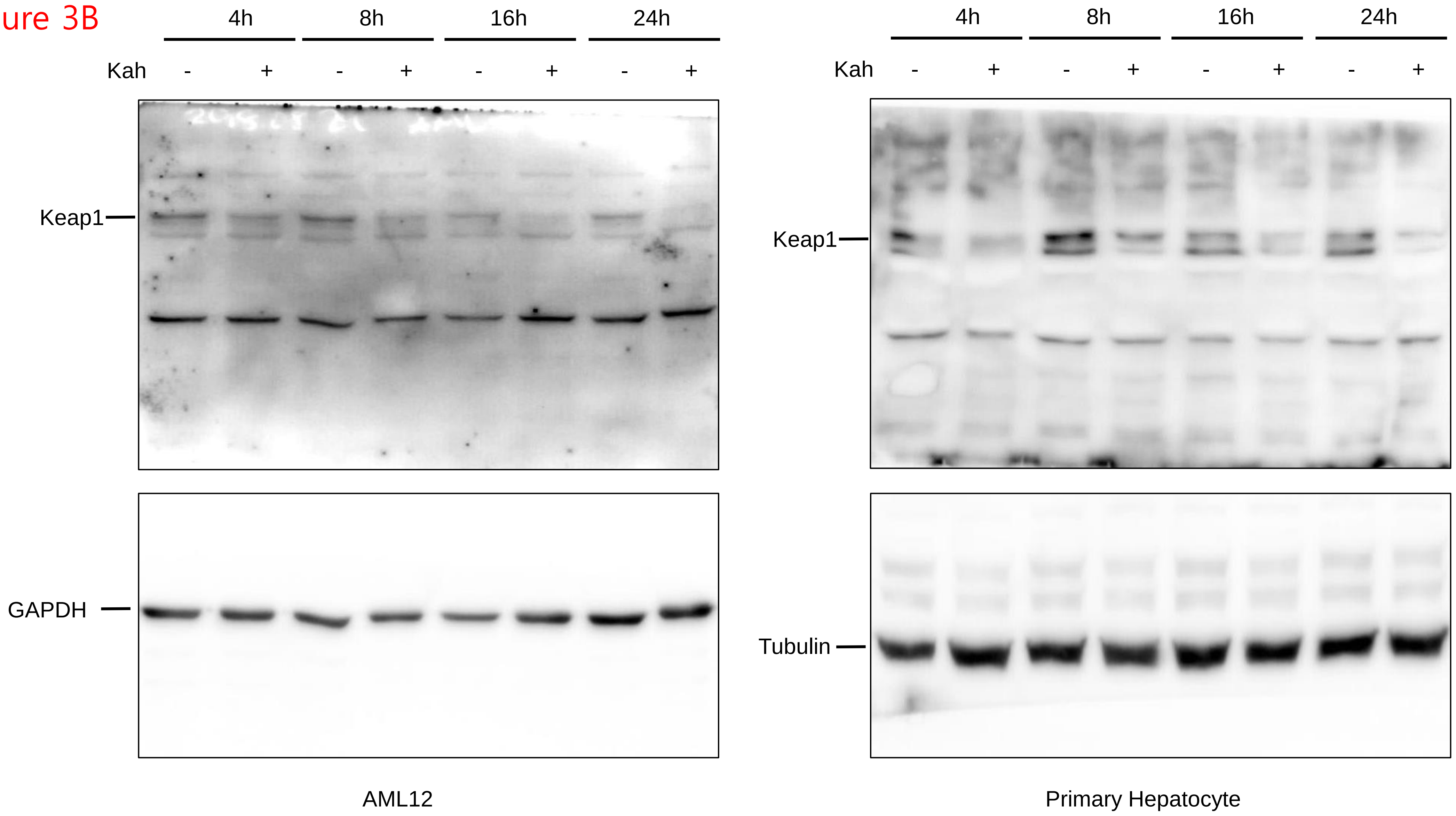
4h
8h
16h
24h
Kah - + - + - + - +
Keap1
GAPDH
AML12
4h
8h
16h
24h
Kah - + - + - + - +
Keap1
Tubulin
Primary Hepatocyte

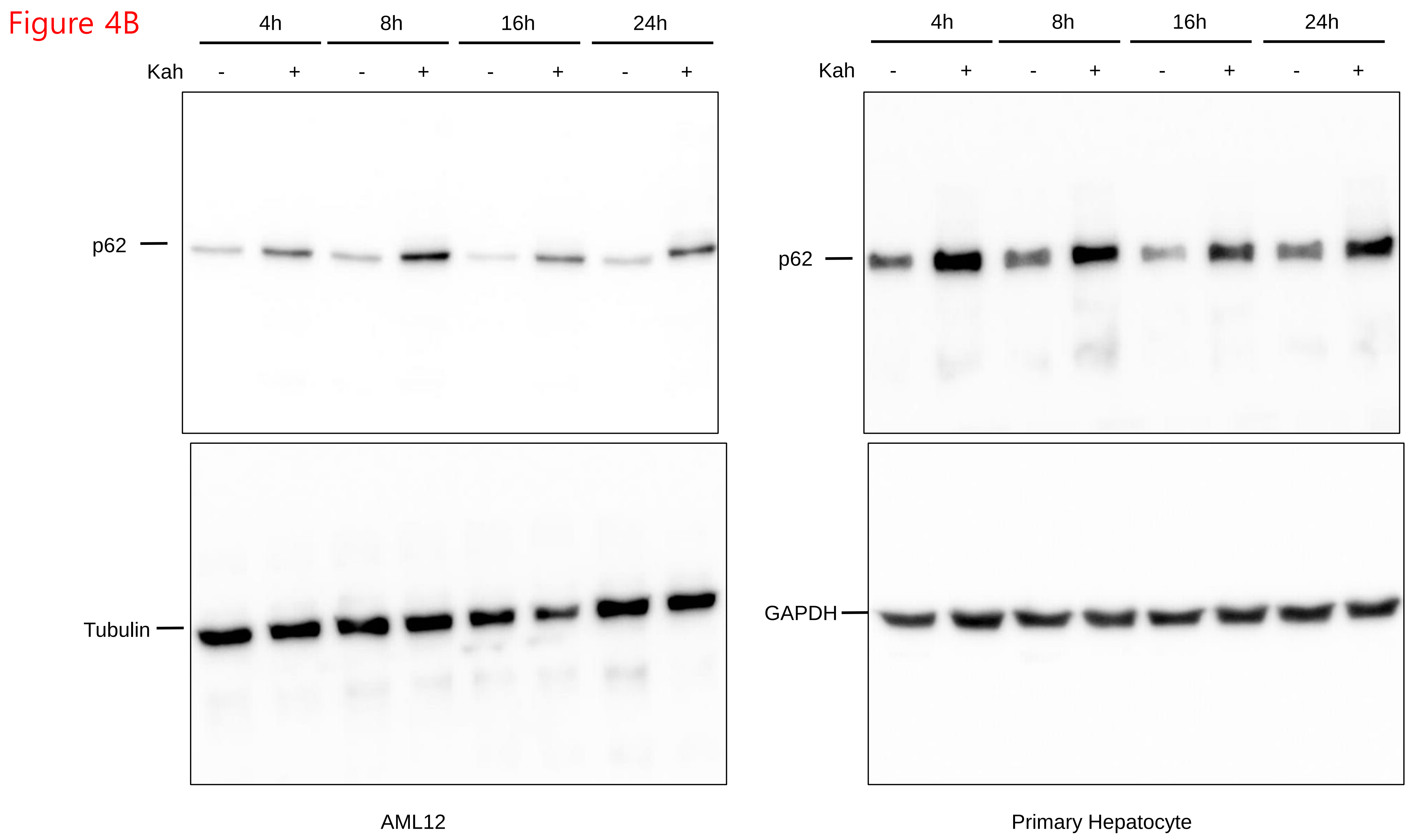
Figure 4B
4h
8h
16h
24h
Kah - + - + - + - +
p62
Tubulin
GAPDH
AML12
Primary Hepatocyte

Figure 4C

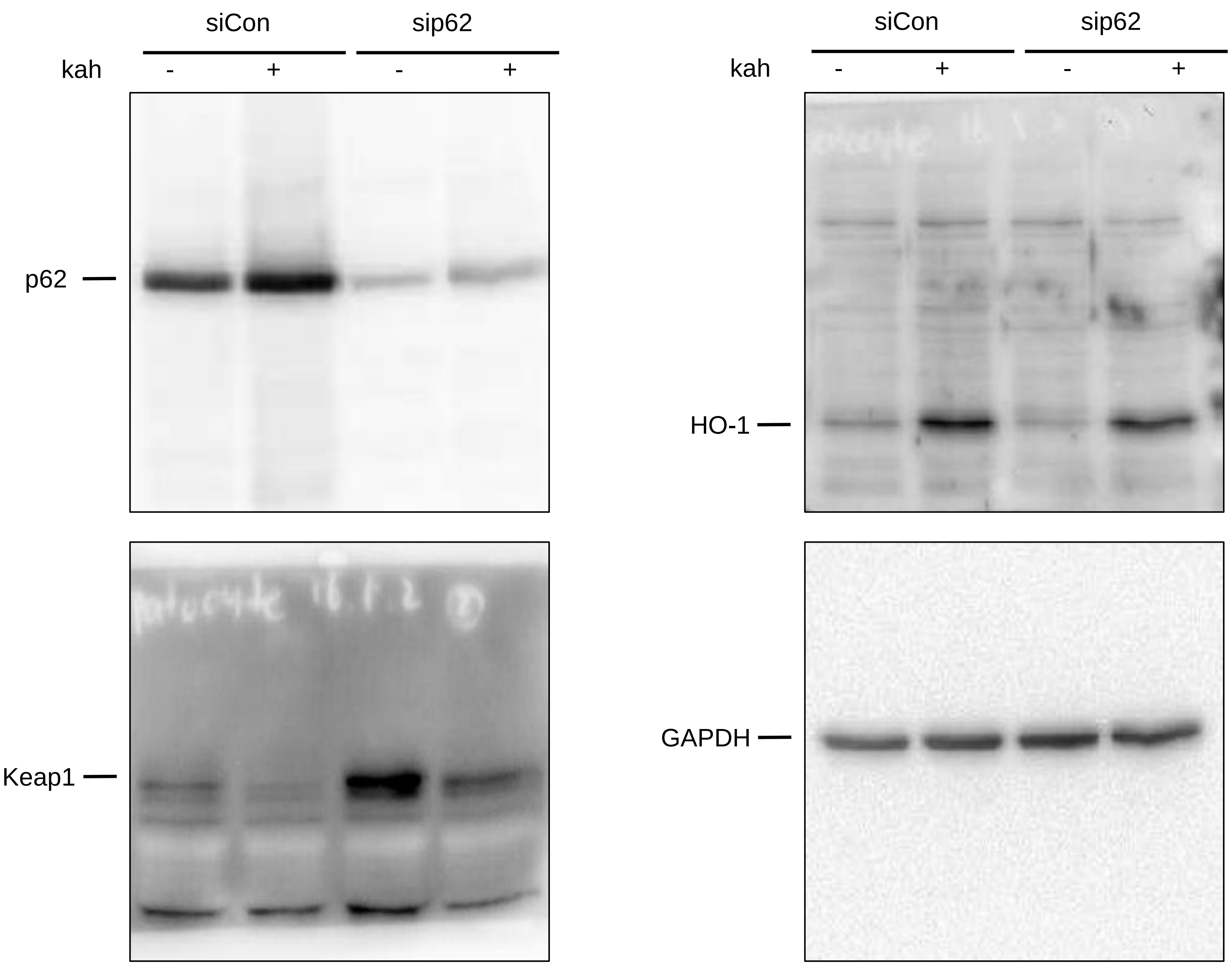

Figure 5A

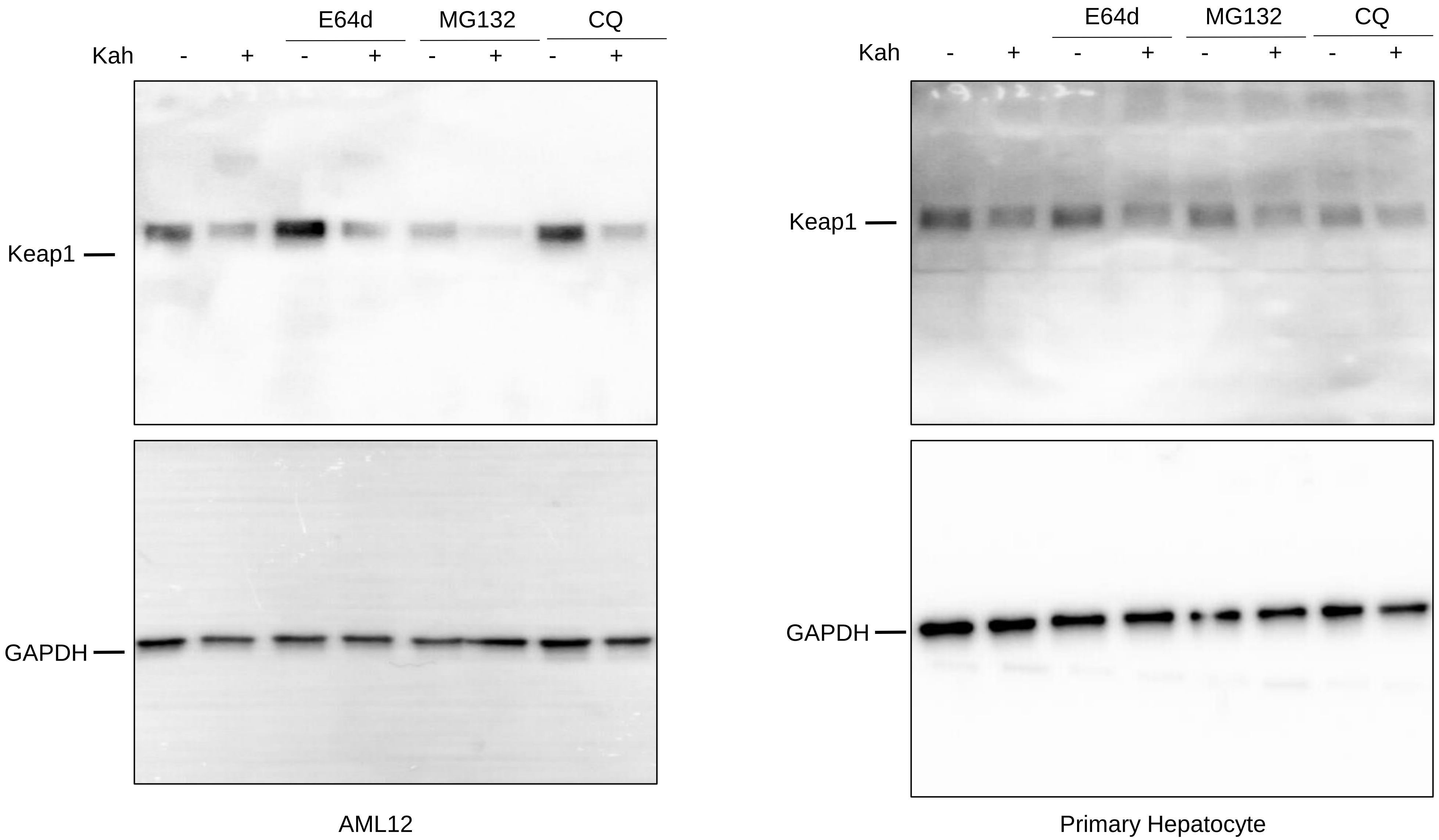
E64d
MG132
CQ
Kah - + - + - + - +
Keap1
GAPDH
AML12
E64d
MG132
CQ
Kah - + - + - + - +
Keap1
GAPDH
Primary Hepatocyte

Figure 5B

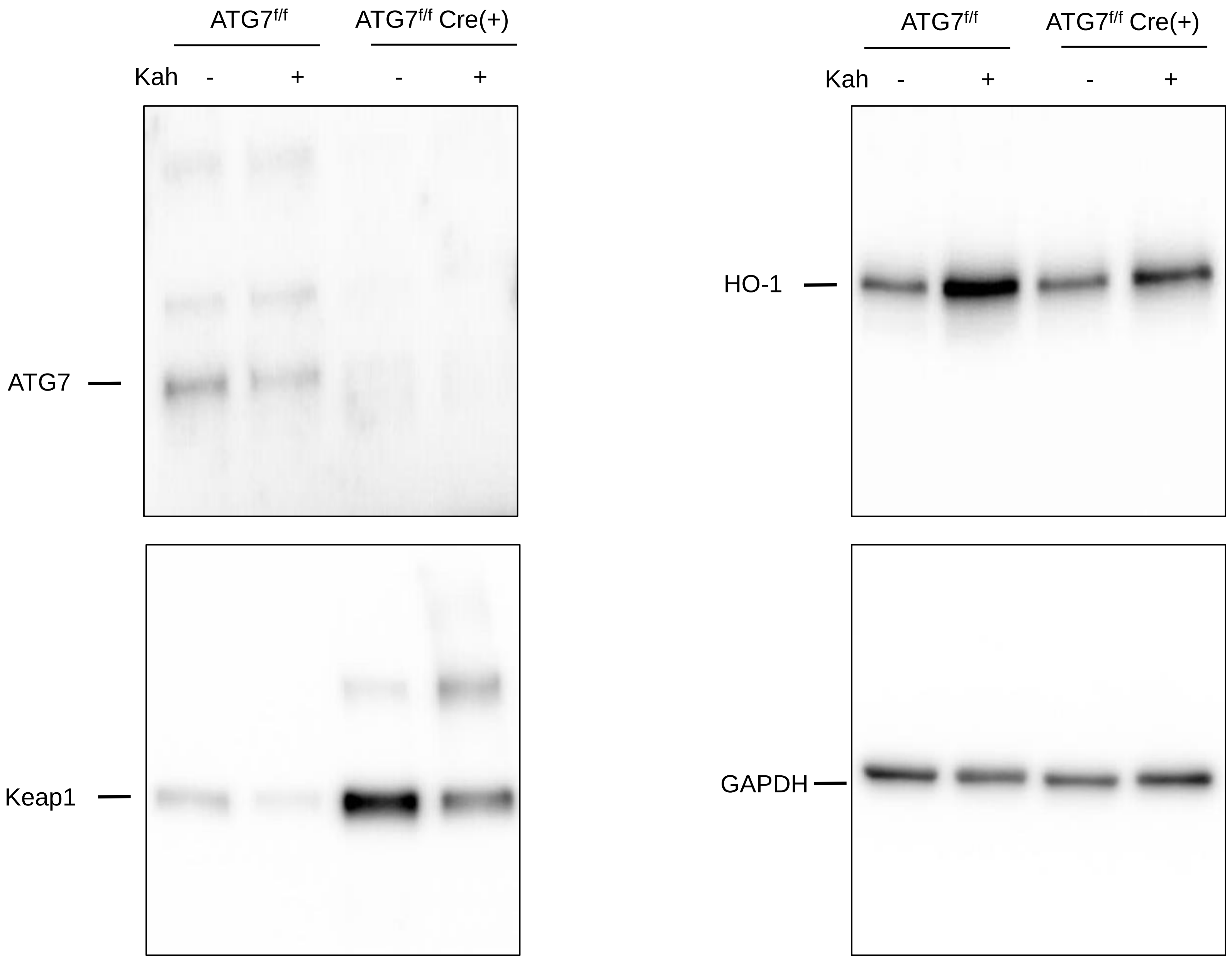

ATG7f/f
ATG7f/f Cre(+)
Kah - + - +
ATG7
Keap1
ATG7f/f
ATG7f/f Cre(+)
Kah - + - +
HO-1
GAPDH

S1 Figure A

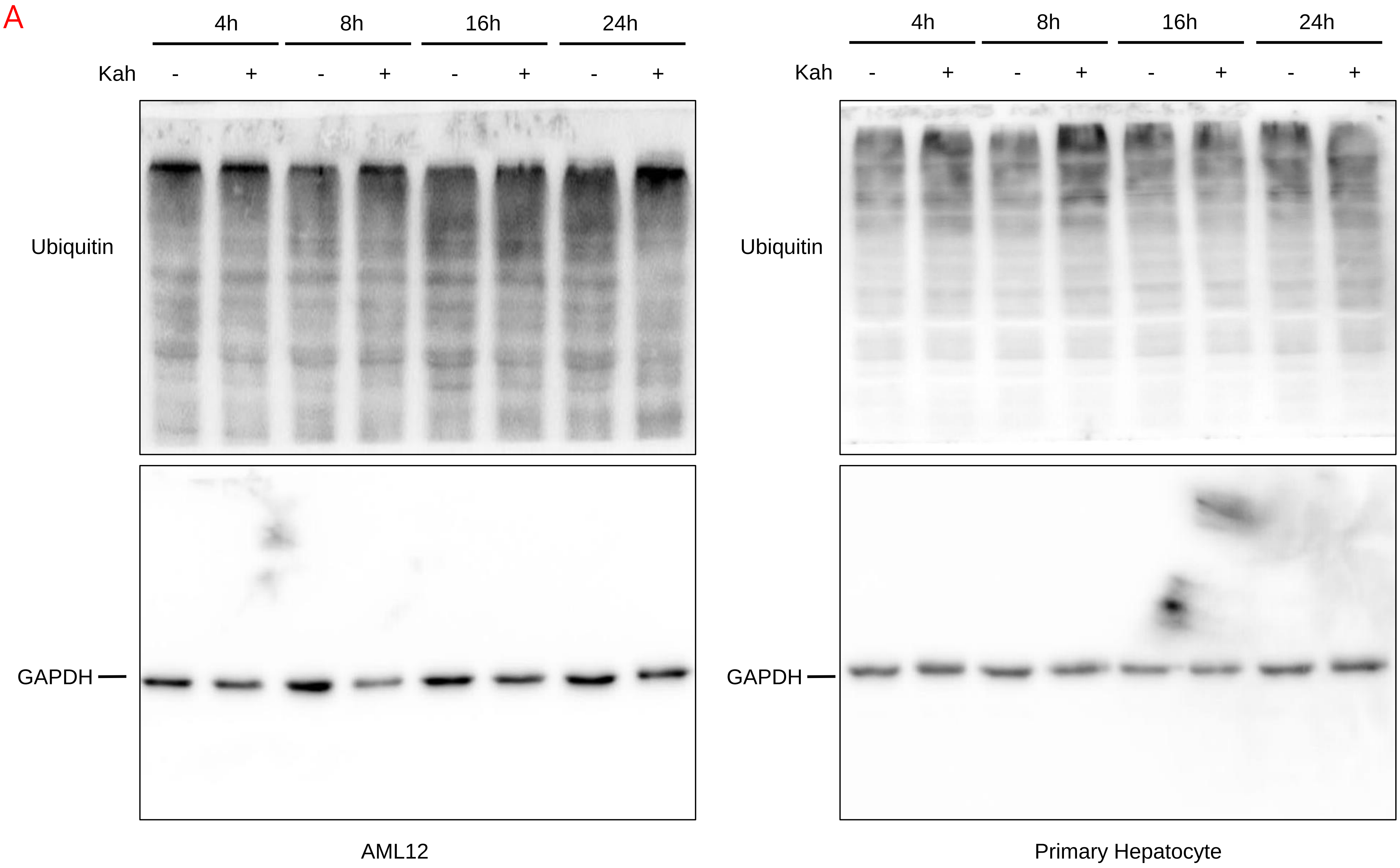

S1 Figure B

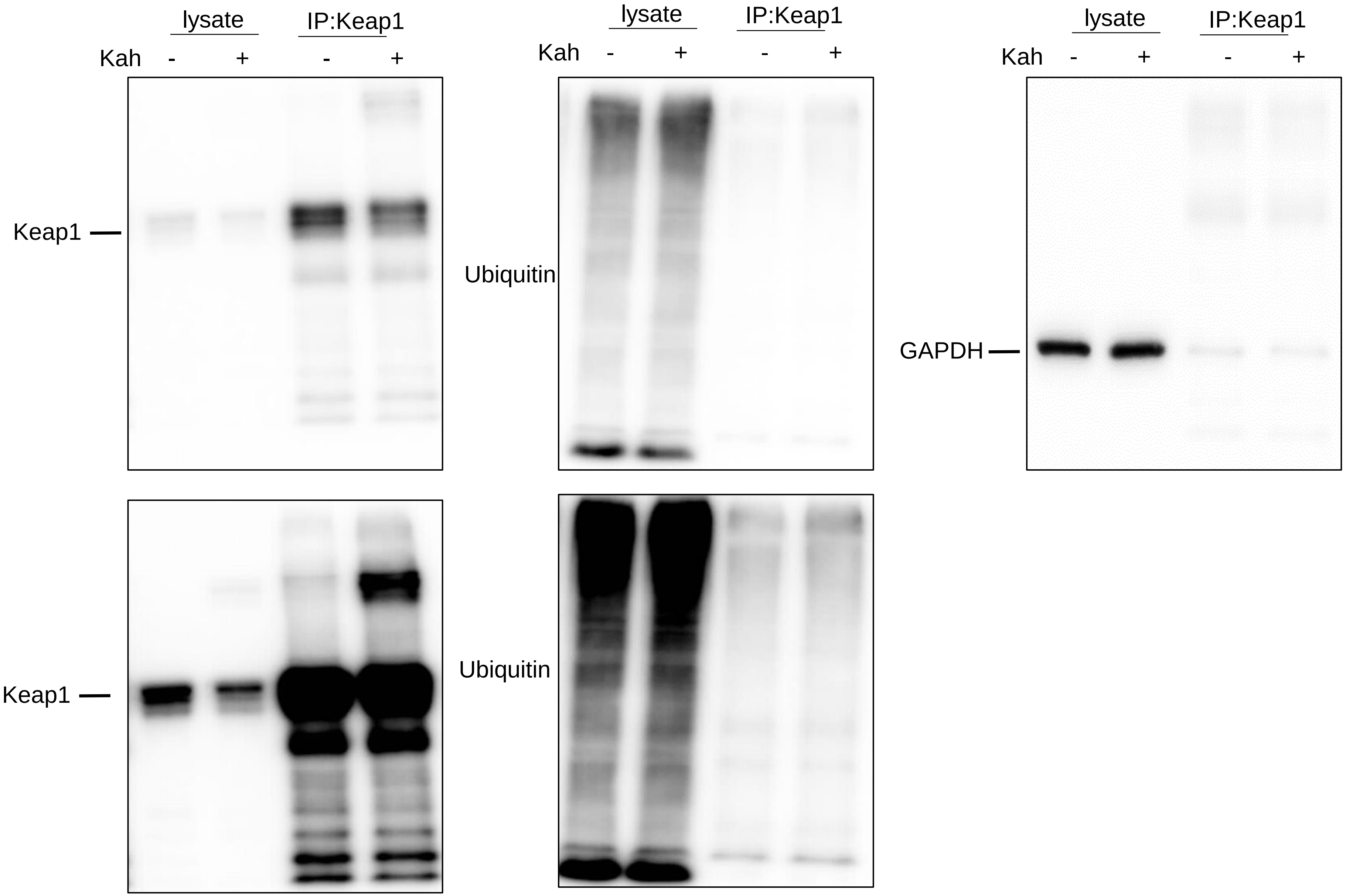

S1 Figure C

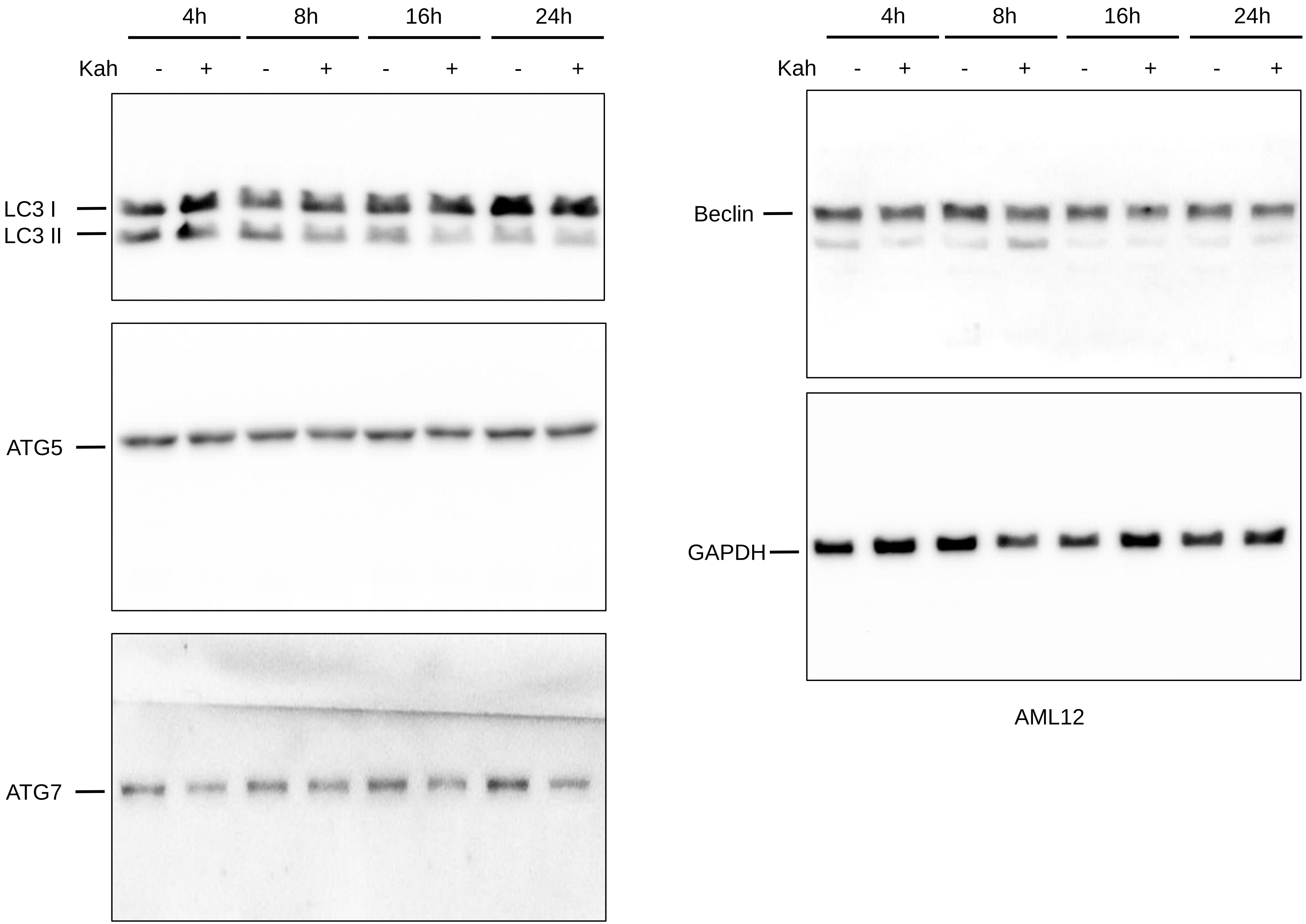
4h
8h
16h
24h
Kah - + - + - + - +
LC3 I
LC3 II
ATG5
ATG7
4h
8h
16h
24h
Kah - + - + - + - +
Beclin
GAPDH
AML12

S1 Figure C

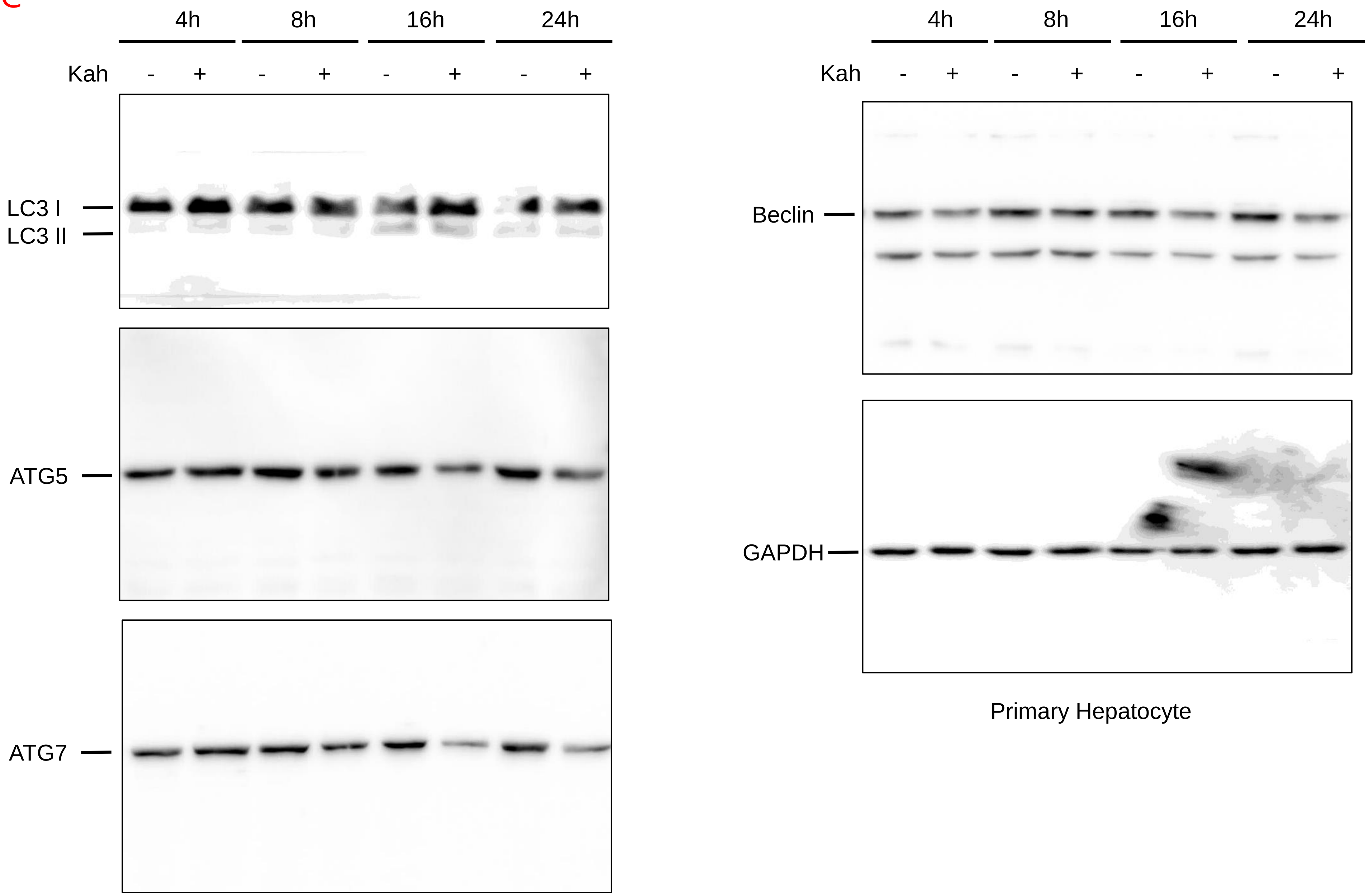
4h
8h
16h
24h
Kah - + - + - + - +
LC3 I
LC3 II
ATG5
ATG7
4h
8h
16h
24h
Kah - + - + - + - +
Beclin
GAPDH
Primary Hepatocyte
